# Parkinson-related parkin reduces α-Synuclein phosphorylation in a gene transfer model

**DOI:** 10.1186/1750-1326-5-47

**Published:** 2010-11-04

**Authors:** Preeti J Khandelwal, Sonya B Dumanis, Li Rebekah Feng, Kathleen Maguire-Zeiss, GW Rebeck, Hilal A Lashuel, Charbel EH Moussa

**Affiliations:** 1 Department of Neuroscience, Georgetown University Medical Center. Washington D.C. U.S.A. 20007; 2 Department of Biochemistry Molecular and Cell Biology. Georgetown University Medical Center. Washington D.C. U.S.A. 20007; 3 Laboratory of Molecular Neurobiology and Neuroproteomics, Brain Mind Institute, Ecole Polytechnique Federale de Lausanne (EPFL), CH-1015 Lausanne, Switzerland

## Abstract

**Background:**

α-Synuclein aggregates in Lewy bodies and plays a central role in the pathogenesis of a group of neurodegenerative disorders, known as "Synucleinopathies", including Parkinson's disease. Parkin mutations result in loss of parkin E3-ubiquitin ligase activity and cause autosomal recessive early onset parkinsonism.

**Results:**

We tested how these two genes interact by examining the effects of parkin on post-translational modification of α-Synuclein in gene transfer animal models, using a lentiviral gene delivery system into the striatum of 2-month old male Sprague Dawley rats.

Viral expression of wild type α-Synuclein caused accumulation of α-Synuclein and was associated with increased cell death and inflammation. α-Synuclein increased PLK2 levels and GSK-3β activity and increased the levels of phosphorylated α-Synuclein and Tau. Parkin co-expression reduced the levels of phosphorylated α-Synuclein and attenuated cell death and inflammation. Parkin reduced PLK2 levels and increased PP2A activation.

**Conclusions:**

These data suggest that parkin reduces α-Synuclein levels and alters the balance between phosphatase and kinase activities that affect the levels of phosphorylated α-Synuclein. These results indicate novel mechanisms for parkin protection against α-Synuclein-induced toxicity in PD.

## Background

Parkinson's disease (PD) is a neurodegenerative disorder characterized by death of dopaminergic neurons in the substantia nigra (SN) and accumulation of α-Synuclein in intracellular inclusions known as Lewy bodies (LBs) [1-10]. LBs are pathological markers of a group of diseases collectively known as "Synucleinopathies" [1,4-6,8,10]. α-Synuclein is natively unfolded and predominantly non-phosphorylated *in vivo *[11], but in aging human brains [12] and Synucleinopathies, a significant fraction of aggregated α-Synuclein is phosphorylated at Ser 129 (p-Ser 129) [11,13]. p-Ser 129 was initially reported to accelerate the oligomerization and fibrillization of α-Synuclein [11,14,15], as well as accumulation and aggregation of α-Synuclein in animal models of Synucleinopathies [16,17]. Paradoxically, recent studies suggest that phosphorylation at Ser 129 inhibits, rather than promotes, α-Synuclein fibrillization [18].

Parkin is an E3-ubiquitin ligase involved in proteasomal degradation of proteins [19]. A loss of function mutation in the parkin gene results in autosomal recessive juvenile PD [20,21]. Specific targets of parkin E3 ubiquitin-ligase activity *in vivo *include an O-glycosylated form of α-Synuclein, α-Synuclein P22 [22], and Pael-R, the parkin-associated endothelin-like receptor *in vitro *[23]. Parkin suppresses the toxicity of both Pael-R *in vitro *[24] and mutated α-Synuclein A53T *in vivo *[25,26]. Parkin deficiency in mice results in accumulation of non-ubiquitinated forms of α-Synuclein in the brain [22,23], and loss of function mutation results in degeneration of dopaminergic neurons in transgenic flies [27]. Although native α-Synuclein does not appear to be a parkin substrate [28], several parkin over-expressing animal models display protection against α-Synuclein toxicity [25,26,29,30], suggesting a link between the two proteins.

Parkin protects against loss of dopaminergic neurons in the rat SN despite the increase in p-Ser 129 [25]. p-Ser 129 is ubiquitinated in LBs [31,32], suggesting that α-Synuclein ubiquitination may be secondary to phosphorylation. Ubiquitinated inclusions are increased in the presence of parkin and synphilin-1 when α-Synuclein is phosphorylated at Ser 129 [33]. To test the potential role of parkin in modulating α-Synuclein post-translational modifications (i.e. ubiquitination and phosphorylation) and toxicity, we used lentiviral gene transfer animal models, which allow us to examine the *in vivo *effects of these proteins.

## Methods

### Cell culture, protein fractionation and Western blot analysis

Human wild type α-synuclein cDNA, a kind gift from Dr. Benoit Giasson, was subcloned into a tetracycline responsive auto-regulated bi-directional expression vector, pBig2i, a kind gift of Dr. Strathdee. The immortalized dopaminergic cell lines, MN9 D were stably transfected with the pBig2isynIRESeGFP as previously reported MN9D_SYN_. MN9 D cells were maintained in Dulbecco's modified Eagle's medium (Sigma, D5648) containing 10% fetal bovine serum (FBS) and hygromycin B (200 μg/mL). Either MN9 D cells or M17 human neuroblastoma cells were plated at a density of 8 × 10^4 ^cells/well for 12-well plates. Synuclein expression was induced with doxycycline (2.0 μg/mL media) in MN9 D cells 24 h prior to infection with multiplicity of infection (m.o.i) of 100 for wild type or mutant T240R lentiviral parkin for an additional 24 h. Human neuroblastoma M17 cells (N = 6) 100 m.o.i of either lentiviral parkin, T240R or LacZ were infected for 24 h. For immunoprecipitation and Western blot analysis, cells were harvested in 1× STEN buffer (50 mM Tris (pH 7.6), 150 mM NaCl, 2 mM EDTA, 0.2% NP-40, 0.2% BSA, 20 mM PMSF and protease cocktail inhibitor), centrifuged at 10,000 × g for 20 min at 4°C and the supernatant containing the soluble fraction of α-synuclein and parkin was collected. Cells were treated with 10 μM okadaic acid (OA) for 3 h to inhibit phosphatases. To extract the insoluble fraction of proteins, the pellet was re-suspended in 4% urea and solublized for Western blot analysis. The soluble fraction of α-synuclein was immunoprecipitated with mouse (1:200) anti-α-synuclein antibody (BD Transduction Laboratories, USA) and phospho-α-synuclein was isolated using a Phospho-Protein Purification Kit (QIAGEN, Cat# 37101).

### Generation of lentiviral constructs

To clone viral constructs used to generate animal models, cDNA templates were cloned into a lentiviral backbone, pLenti6/-D-TOPO (Invitrogen, CA, USA), using Directional TOPO cloning kit. Human wild type parkin was amplified from pcDNA3.1 plasmids (gift from Dr Ted Dawson, Johns Hopkins University, Baltimore) by PCR using 5'-CACC CCA TGA TAG TGT TTG TCA GGT TC-3' as a forward primer and 5'-GTT GTA CTT TCT CTT CTG CGT AGT GT-3' as a reverse primer. Wild type α-Synuclein was amplified from pCDNA3.1(+)-Syn_wt _(Gift from Dr. Benoit Giasson) vector by PCR using 5'-CAC CAT GGA TGT ATT CAT GTT TCC-3' as a forward primer and 5'-GGC TTC AGG TTC GTA GTC TTG AT-3' as a reverse primer. Lentiviral constructs were packaged with ViraPower™ Lentiviral Expression Systems (Invitrogen) and titrated in human neuroblastoma M17 cells.

### Stereotaxic injection

Stereotaxic surgery was performed to inject the lentiviral constructs encoding parkin, LacZ or α-Synuclein into the striatum of 2-month old male Sprague-Dawley rats (total N = 16) weighing between 170-220 g as indicated in [34]. The stereotaxic coordinates for the striatum were 3 mm lateral, 6 mm ventral, 0.48 mm posterior. Animals (N = 4 per group) were injected into one [35] side of striatum with 1) a lentiviral-LacZ vector at 2 × 10^10 ^m.o.i,; 2) with 1 × 10^10 ^m.o.i lentiviral-parkin and 1 × 10^10 ^m.o.i lentiviral-LacZ; 3) 1 × 10^10 ^m.o.i lentiviral-α-Synuclein and 1 × 10^10 ^m.o.i lentiviral-LacZ; or 4) and 1 × 10^10 ^m.o.i lentiviral-α-Synuclein and 1 × 10^10 ^m.o.i lentiviral-parkin. Animals were euthanized 4 weeks post-injection. All animal work was approved by Georgetown University Animal Care and Use Committee (GUACUC), under protocol # 07-021.

### Immunohistochemistry of brain sections

Immunohistochemistry was performed on 20 micron-thick brain sections. Parkin was probed with anti-parkin (1:200) mouse (PRK-8) monoclonal antibody (Signet Labs, Dedham, MA). α-Synuclein was probed (1:200) with mouse antibody (BD Transduction Laboratories, USA), or (1:200) human specific mouse monoclonal antibody (Thermo Fisher) followed by DAB staining. GFAP was probed (1:200) with monoclonal antibody (Millipore Corporation, USA), and microglia was probed (1:200) with IBA-1 polyclonal antibody (Wako, USA). Further staining was performed to assess neural disintegrative-degeneration in animal models using FD NeuroSilver™ staining kit II (FD NeuroTechnologies, Inc, Baltimore, MD), which provides high contrast and rapid silver staining for the microscopic detection of neuronal and fiber degeneration *in vivo*.

*Stereological methods counting *were applied by a blinded investigator using unbiased stereology analysis (Stereologer, Systems Planning and Analysis, Chester, MD) to determine the total positive cell counts in 20 striatal fields on at least 10 brain sections (~400 positive cells per slide) from each animal (N = 4). An optical fractionator sampling method was used to estimate the total number of positive cells with multi-level sampling design. Cells were counted within the sampling frame determined optically by the fractionator and cells that fell within the counting frame were counted as the nuclei came into view while focusing through the *z*-axis. Striatal TH-positive staining was assessed by optical density (OD) measurements. Using an Optronics (Goleta, CA) digital camera and a constant illumination table, digitalized images of TH immunostained striatal sections were collected. ODs were measured using Image-Pro Plus software (Version 3.0.1; Media Cybernetics, Silver Spring, MD). The OD was measured from 6 striatal coronal sections and the final reading was calculated as an average of those values. Nonspecific background correction in each section was done by subtracting the OD value of the corpus callosum from the striatal OD value obtained from the same section. The OD analysis was performed under blinded condition.

### Western blot analysis

Two weeks post-injection, the ipsilateral striatum was isolated from the contralateral one and brain tissues were homogenized in 1× STEN buffer (50 mM Tris (pH 7.6), 150 mM NaCl, 2 mM EDTA, 0.2% NP-40, 0.2% BSA, 20 mM PMSF and protease cocktail inhibitor), centrifuged at 10,000 × g for 20 min at 4°C and the supernatant containing the soluble protein fraction was collected. The supernatant was analyzed by Western blot on SDS NuPAGE 4-12% Bis-Tris gel (Invitrogen). Protein estimation was performed using BioRad protein assay (BioRad Laboratories Inc, Hercules, CA). Parkin was immunoprobed with (1:1000) mouse anti-parkin (PRK8) antibody (Signet Labs, Dedham, MA) and α-Synuclein was immunoprobed with (1:1000) mouse anti-α-Synuclein antibody (BD Transduction Laboratories, USA). Phospho-serine 129 (p-Ser 129) α-Synuclein was immunoprobed (1:500) with rabbit p-Ser 129 antibody (Affinity Bio-Reagents, USA), and p-Ser 87 was immunoprobed (1:100) with rabbit anti-p-Ser 87 antibody (Gift from Dr. Hilal Lashuel, Ecole Polytechnique Federale de Lausanne, Switzerland). β-actin was probed (1:1000) with polyclonal antibody (Cell Signaling Technology, Beverly, MA, USA). TNF-α was probed with (1:1000) anti-TNF-α rabbit antibody (Serotec), iNOS was probed with (1:1000) anti-iNOS rabbit antibody (BD Transduction Laboratories, USA), and tyrosine hydroxylase (TH) was probed (1:1000) with rabbit antibody (Millipore, Temecula, CA). Total GSK-3β was probed (1:1000) with monoclonal antibody (Biosource, Carlsbad, CA, USA) and p-GSK-3β at tyrosine 216/279 was probed (1:1000) with polyclonal antibody (Biosource, Carlsbad, CA, USA), PLK-2 was probed with (1:1000) anti-PLK2 antibody (ABNOVA). Total tau was probed (1:1000) with tau-15 monoclonal antibody (Chemicon, Temecula, CA, USA), and phosphorylated tau was probed (1:1000) with epitopes against polyclonal serine-396 (Chemicon, Temecula, CA, USA), polyclonal AT8 (1:1000) Serine-199/202 (Biosource, Carlsbad, CA, USA), polyclonal (1:1000) serine-262 (Affinity Bio-Reagents, USA), AT180 polyclonal (1:1000) threonine-231 (Biosource, Carlsbad, CA, USA), AT270 polyclonal (1:1000) Threonine-181 (Biosource, Carlsbad, CA, USA). Phosphatase 2A subunits A, B and C sub-units were probed (1:1000) with polyclonal antibodies (Thermo Scientific, USA). Western blots were quantified by densitometry using Quantity One 4.6.3 software (Bio Rad). At least N = 4 was used in each group and the data was analyzed as mean ± StDev and statistical comparison of variables was obtained by ANOVA with Neumann Keuls multiple comparison test, P < 0.05.

### Phosphatase activity assay

To measure phosphatase activity, we used Malachite Green Phosphate detection kit (R&D Systems) and performed the assay on triplicates of striatal brain extracts in 96-well dishes. The absorbance was read at 620 nm according to the manufacturers' protocols.

### Caspase-3 fluorometric activity assay

To measure caspase-3 activity in the animal models, we used EnzChek^® ^caspase-3 assay kit #1 (Invitrogen, Molecular Probes, Inc) on striatal extracts and Z-DEVD-AMC substrate and read the absorbance was read according to manufacturer's protocol.

### Graphs and statistics

All graphs and statistical analyses were performed in GraphPad Prism Software (GraphPad Prism Software, Inc. CA. USA). All statistics were performed using ANOVA with Newman Keuls multiple comparison test and P < 0.05 as statistically significant, N = 4 for animals and N = 6-12 for cell culture studies.

## Results

### Lentiviral α-Synuclein and parkin expression in gene transfer animal models

**Figure 1:**
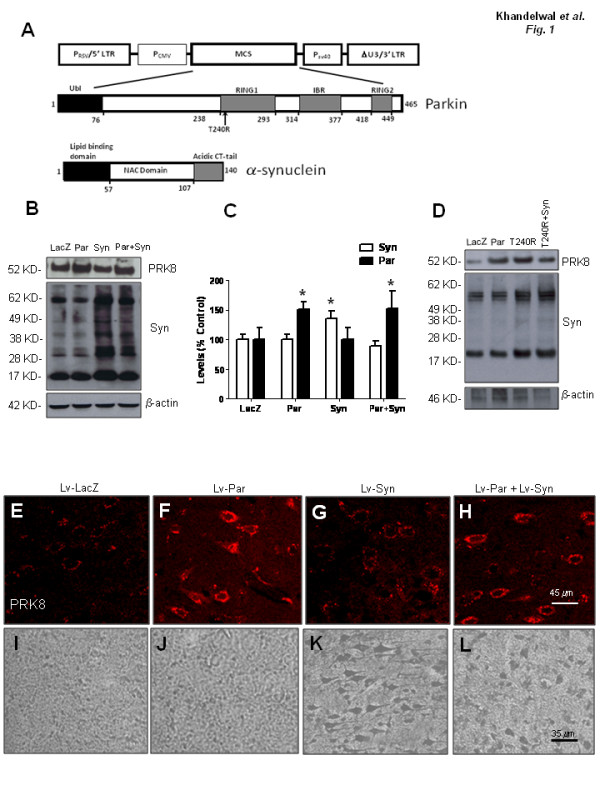
**A). Generation of α-Synuclein gene transfer animal models and parkin effects on total α-Synuclein levels**. Schematic representation of lentiviral clones encoding human α-Synuclein and human wild type and mutant T240R parkin. B). Western blot analysis of striatal brain extracts using 4-12% NuPAGE SDS gel (Invitrogen) probed with parkin (PRK8), and α-Synuclein antibodies and C). Densitometric analysis. D). Western blot analysis of M17 cell extracts infected with T240R mutant parkin and human α-Synuclein using 4-12% NuPAGE SDS gel (Invitrogen) probed with parkin (PRK8), and α-Synuclein antibodies. E). Parkin immnunostaining (PRK-8) of 20 micron-thick rat striatal sections injected with E). lentiviral LacZ (Lv-LacZ), F). lentiviral parkin (Lv-Par), G). Lentiviral α-Synuclein (Lv-Syn) and H). Lentiviral parkin+lentiviral α-Synuclein (Lv-Par+Lv-Syn). Immunoreactivity using human anti-α-Synuclein antibody followed by DAB staining in sections of rat striatum injected with I). lentiviral LacZ, J). lentiviral parkin, K). lentiviral α-Synuclein and L). lentiviral parkin+α-Synuclein. Asterisk is significantly different to LacZ control, ANOVA, with Neumann Keuls multiple comparison N = 4, P < 0.05.

To generate gene transfer animal models and test the effects of human parkin and α-Synuclein *in vivo*, we injected either lentiviral parkin (Lv-Par; Figure 1A) or α-Synuclein (Lv-Syn; Figure 1A) or LacZ (Lv-LacZ) or both (Lv-Par + Lv-Syn) into the striatum of 2-month old male Sprague Dawley rats. To test the effects of parkin on α-Synuclein, we analyzed infected striatal extracts 4 weeks post-injection by Western blot with parkin and α-Synuclein antibodies. As expected, the level of parkin was significantly (ANOVA, Neumann Keuls, P < 0.05) increased (~50%, N = 4) in animals injected with parkin (Figure 1B &1C), and α-Synuclein was significantly increased (41%, N = 4) in α-Synuclein lentivirus injected brains without parkin expression. Parkin and α-Synuclein co-expression showed increased parkin levels, but reduced total α-Synuclein to LacZ levels (Figure 1B &1C). Higher molecular weight bands (>17 KD) were also observed in striatal rat brain samples. To ascertain that wild type parkin had an effect on α-Synuclein levels, we co-infected human M17 neuroblastoma cells with mutant T240R parkin and α-Synuclein. Both human α-Synuclein and T240R were over-expressed (Figure 1D) but mutant parkin had no effects on α-Synuclein levels. We also tested the expression levels of parkin and human α-Synuclein by immunohistochemistry, and observed an increase in parkin immunoreactivity in parkin (Figure 1F; Lv-Par), and parkin+α-Synuclein (Figure 1H; Lv-Par+Lv-Syn) injected brains compared to LacZ (Figure 1E; Lv-LacZ) and α-Synuclein (Figure 1G, Lv-Syn) injected brains. We then used a human anti-α-Synuclein antibody to examine the expression level of lentiviral α-Synuclein. No expression of human α-Synuclein was observed in LacZ (Figure 1I) and parkin expressing brains (Figure 1J). Injection of lentiviral α-Synuclein caused cellular accumulation of human α-Synuclein (Figure 1K), but this accumulation was reduced with parkin co-expression (Figure 1L). These data suggest that parkin reduces the expression levels of α-Synuclein.

### The effects of lentiviral parkin expression on α-Synuclein-induced cell death in gene transfer animal models

**Figure 2:**
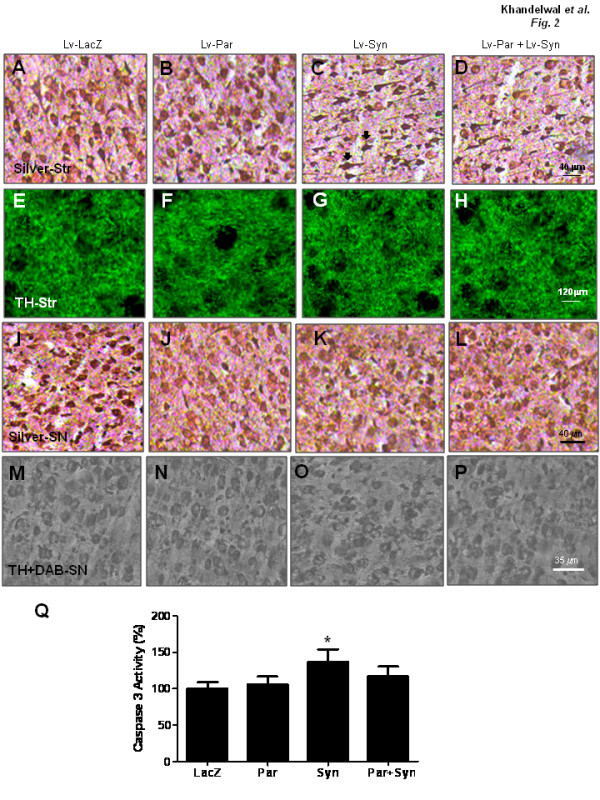
**α-Synuclein-induced cell death and parkin protection in gene transfer animal models**. Silver staining in sections of rat striatum injected with A). lentiviral LacZ, B). lentiviral parkin and C). lentiviral α-Synuclein (arrows indicate silver-positive cells) and D). lentiviral parkin+α-Synuclein. Sections of rat striatum stained with TH antibody in lentiviral lacZ (Figure 2E), parkin (Figure 2F), α-Synuclein (Figure 2G) and parkin+α-Synuclein (Figure 2H) injected brains. Silver staining in 20 micron thick sections of rat SN from brains injected with I). lentiviral LacZ, J) parkin, K). α-Synuclein and L). parkin+α-Synuclein. TH staining of SN neurons from brains injected with M). lentiviral LacZ, N) parkin, O). α-Synuclein and P). parkin+α-Synuclein. Q). Histogram represents caspase-3 activity assay. Asterisk is significantly different to LacZ control, ANOVA, with Neumann Keuls multiple comparison N = 4, P < 0.05.

To examine whether the lentivirus, α-Synuclein or parkin cause cell death in these animal models, we used silver staining to detect cell death and degeneration *in vivo*. Injection of lentiviral LacZ into the striatum did not result in detectable positive silver staining (Figure 2A) indicating that the lentivirus does not cause cell death in our control animals. No silver-positive cells were detected in the parkin injected striatum (Figure 2B), but silver-positive cells were observed when α-Synuclein was expressed (Figure 2C, arrows). Morphological changes were also observed in silver-positive (dark) cells. Stereological counting demonstrated a significant increase (32%, N = 4) in silver-positive cells in α-Synuclein injected striatum (Figure 2C) compared to LacZ injection (Figure 2A). Co-expression of parkin and α-Synuclein showed fewer (13% decrease of control, N = 4) silver-stained cells (Figure 2D) compared to α-Synuclein expression alone (Figure 2C). We performed tyrosine hydroxylase (TH) staining to examine whether cell death in striatal neurons was accompanied by loss of dopaminergic nerve terminals. TH staining revealed no noticeable differences between LacZ (Figure 2E), parkin (Figure 2F), α-Synuclein (Figure 2G) and parkin+α-Synuclein (Figure 2H) injected brains. Optical density measurements of TH-positive staining of dopaminergic terminals in the striatum did not show a statistically significant (16% decrease) difference between α-Synuclein injected brains and LacZ, parkin or parkin+α-Synuclein injected brains, suggesting no loss of dopaminergic nerve terminals in the striatum.

Further examination of the SN revealed no significant changes in silver staining between the different treatments (Figure 1I-L), indicating no degenerative loss of neurons in SN. Further analysis with TH staining of dopamine neurons in SN also showed no noticeable differences between α-Synuclein (Figure 2O) injected brains and LacZ (Figure 2M), parkin (Figure 2N) and parkin+α-Synuclein (Figure 2P).

To further test the effects of parkin and α-Synuclein expression of neuronal cell death, we performed caspase-3 activity assays in striatal brain extracts and observed a significant increase (34%, N = 4) in caspase-3 activity in α-Synuclein (Figure 2Q) expressing brains compared to LacZ or parkin. Parkin expression reduced caspase-3 activity back towards control level, suggesting that parkin may protect against α-Synuclein-induced activation of caspase-3.

### Parkin expression reduces α-Synuclein-induced inflammation

**Figure 3:**
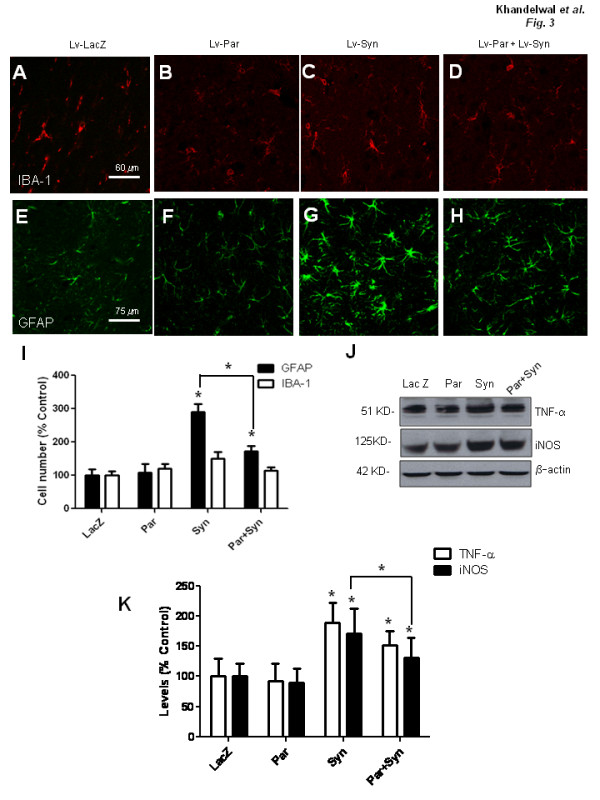
**α-Synuclein-induced neuro-inflammation and parkin protection**. Immunostaining with microglia IBA-1 antibody in 20 micron thick striatal sections of rat brains injected with A). lentiviral LacZ (Lv-LacZ), B). parkin (Lv-Par), C). α-Synuclein (Lv-Syn) and D). Lentiviral parkin+α-Synuclein (Lv-Par+Lv-Syn). Immunostaining with astrocytic GFAP in rat striatum injected with E). lentiviral LacZ, F). parkin, G). α-Synuclein and H). Lentiviral parkin+α-Synuclein I). Histograms represent stereological counting of IBA-1 and GFAP positive cells. J). Western blot of TNF-α and iNOS in striatal extracts analyzed on 10% NuPAGE SDS gel (Invitrogen) and I). Densitometric analysis of immunoblots, Asterisk is significantly different to LacZ control or as indicated, ANOVA, with Neumann Keuls multiple comparison N = 4, P < 0.05.

To test whether α-Synuclein expression induces inflammation in our animal model, we examined signs of glial activation. No noticeable differences in the morphology or number of microglia were detected with IBA-1 immunostaining (Figure 3A-D). Injection with lentiviral LacZ does not result in any changes to either microglial (Figure 3A) or astrocytic (Figure 3E) morphology or number. Astrocyte staining with glial fibrillary acidic protein (GFAP) showed a marked change in cell morphology (Figure 3G) and a significant increase (~190%, N = 4) in glial number (P < 0.05) in α-Synuclein brains (Figure 3G &3I) compared to LacZ (Figure 3E &3I) and parkin injected brains (Figure 3F &3I). Parkin co-expression with α-Synuclein significantly reversed the effects of α-Synuclein on astrocyte number (~50%, N = 4) and morphology, but not to control level (Figure 3H &3I). Further assessment of inflammatory molecules by Western blot revealed a significant increase in TNF-α (~100%, N = 4) and iNOS (71%) in the ipsilateral striatum (P < 0.05) in α-Synuclein injected brains compared to LacZ or parkin injected brains (Figure 3J &3K). Parkin partially reversed the effects (~20% increase) of α-Synuclein on TNF-α levels (Figure 3J &3K), and significantly (P < 0.05) reduced the α-Synuclein effects on iNOS (40%, N = 4). These data demonstrate that α-Synuclein causes inflammation in the rat striatum and parkin expression reverses these inflammatory reactions, consistent with the effects of parkin on neuronal degeneration.

### The effects of human α-Synuclein and parkin on modification of endogenous Tau

**Figure 4:**
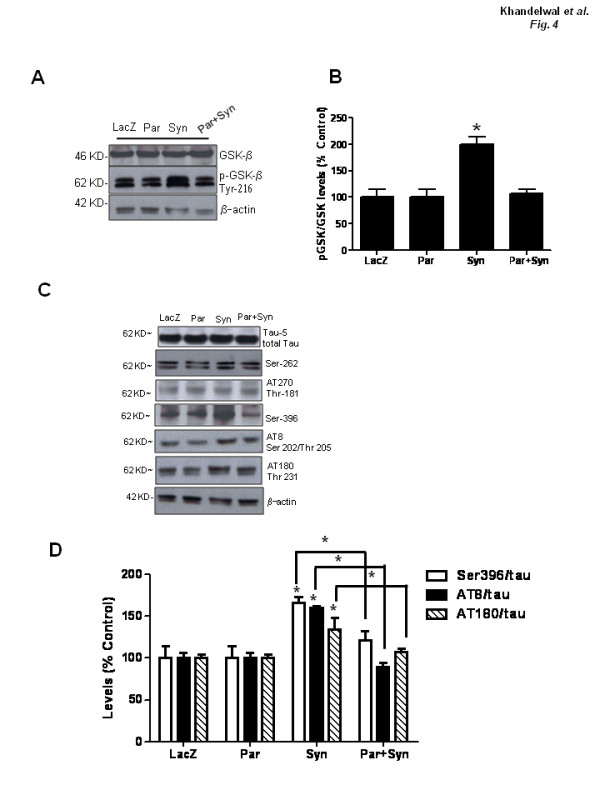
**α-Synuclein-induced activation of GSK-3β and Tau hyper-phosphorylation and parkin protection**. A). Western blot analysis of striatal brain extracts on Nu-PAGE SDS 10% gel (Invitrogen) and B). Densitometry analysis showing GSK-3β activity. C). Western blot analysis showing different Tau epitopes and. B) densitometric analysis of immunoblots, Asterisk is significantly different, ANOVA, with Neumann Keuls multiple comparison N = 4, P < 0.05.

To test the effects of lentiviral gene delivery on kinases and Tau phosphorylation, striatal extracts were analyzed using Western blot 4 weeks post-injection. Human α-Synuclein resulted in a significant increase in phospho-GSK-3β (100%, ANOVA, P < 0.05, N = 4), indicating an increase in the kinase activity in α-Synuclein injected brains (Figure 4A &4B). However, human parkin completely reversed the effects of α-Synuclein on GSK-3β activation back to LacZ (control) levels in the rat brain. Neither parkin nor LacZ nor α-Synuclein had any effects on AKT activation (data not shown). To examine changes in Tau phosphorylation, we probed for different Tau epitopes (Figure 4). No changes in the levels of total Tau or Tau phosphorylation at Ser 262 and Thr 181 (Figure 4C) were observed. However, significant increases (ANOVA, P < 0.05, N = 4) were detected on other Tau phosphorylation sites, including Ser 396 (34%), Ser 202/Thr 205 (38%, N = 4) and Thr 231 (19%, N = 4) in α-Synuclein injected brains (Figure 4C &4D). Parkin significantly reversed the effects of α-Synuclein on Ser 202/Thr 205 and Thr 231 and Ser 396 (Figure 4C &4D) back to LacZ levels. These data indicate that α-Synuclein over-expression increases GSK-3β activity and endogenous Tau hyper-phosphorylation and parkin reverses these effects.

### Parkin decreases α-Synuclein phosphorylation

**Figure 5:**
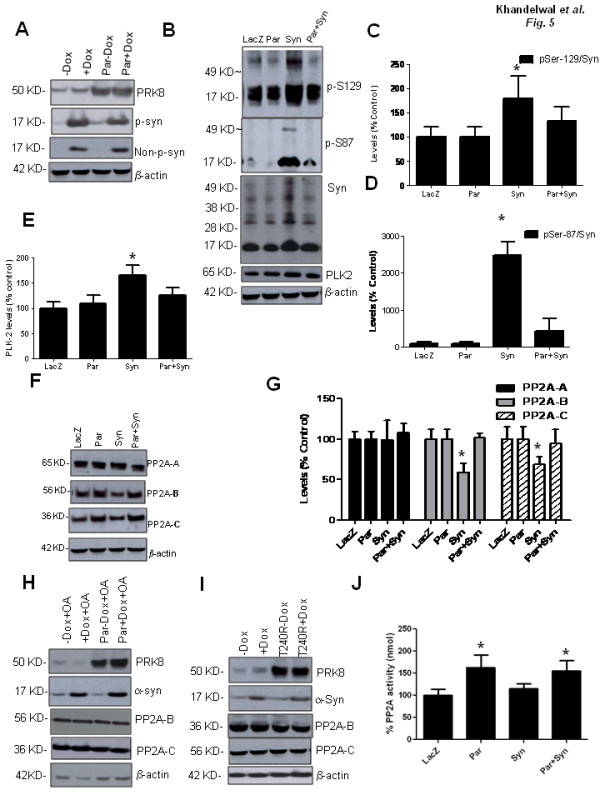
**Parkin expression prevents α-Synuclein phosphorylation and increases phosphatase activity**. A). WB of fractionated α-Synuclein from α-Synuclein stably transfected MN9 D cells and infected with lentiviral parkin and B). WB analysis of striatal brain extracts using 4-12% NuPAGE SDS gel (Invitrogen) probed with various α-Synuclein antibodies and PLK2 antibody. C, D&E). Densitometry of blots expressed as % control. F). WB analysis of protein phosphatase-2A subunits from striatal brain extracts and. G). Densitometry of blots expressed as % control. H). WB of α-Synuclein stably transfected MN9 D cells infected with lentiviral parkin and treated with PP2A inhibitor okadaic acid, and I) WB of T240R mutant and PP2A subunits. J). Phosphatase activity assay. Asterisk **is **significantly different, ANOVA with Neumann Keuls multiple comparison N = 4 for animals, N = 6 for MN9 D cells, P < 0.05.

To study the effects of parkin on α-Synuclein phosphorylation, we expressed parkin in doxycycline-inducible α-Synuclein MN9 D cells. Lentiviral parkin expression resulted in a significant (50%, N = 12) increase in parkin level (Figure 5A) compared to non-transfected cells. Fractionation of total α-Synuclein into phosphorylated (p-α-Syn) and non-phosphorylated α-Synuclein (non-p-α-Syn) showed a significant increase of p-α-Synuclein expression levels in +Dox cells (Figure 5A) compared to -Dox cells. The level of non-p-α-Synuclein in -Dox cells was undetectable by Western blot. Cells expressing parkin demonstrated a significant decrease (30%, N = 12) in p-α-Synuclein (Figure 5A), and no change in the level of non-p-α-Synuclein (Figure 5A), suggesting that the change in total α-Synuclein levels may be due to changes in phosphorylated α-Synuclein fraction.

We then tested whether parkin affects α-Synuclein phosphorylation *in vivo*. Striatal extracts of lentiviral parkin and/or α-Synuclein injected brains were analyzed 4 weeks post-injection, with parkin and α-Synuclein antibodies. No difference in p-Ser 129 was detected between LacZ and parkin injected brains (Figure 5B &5C). The ratio of p-Ser 129/total α-Synuclein (p-Ser129/Syn) was significantly (72%, N = 4) increased (P < 0.05) (Figure 5B &5C), and higher molecular weight species (~49 Kd) were observed in the α-Synuclein injected brains. Co-expression of parkin and α-Synuclein resulted in a significant decrease (P < 0.05, N = 4) in the ratio of p-Ser129/Syn (32%, N = 4) and a decrease in the levels of higher molecular weight species. Endogenous p-Ser 87 was not detected in the parkin expressing rat brain (Figure 5B), but p-Ser 87/Syn was significantly elevated (2350-fold, N = 4) in brains injected with human α-Synuclein. Parkin significantly reduced the level of p-Ser 87 (Figure 5B &5D) when it was co-expressed with α-Synuclein. A higher molecular weight size band (~49 Kd) was observed in brains expressing human α-Synuclein when probed with the p-Ser 87 antibody (Figure 5B). These data indicate that parkin over-expression protects against α-Synuclein-induced pathology and reduces the levels of human p-Ser 87 and p-Ser 129 *in vivo*. We further tested the levels of Polo-Like-Kinase-2 (PLK2), which is known to phosphorylate α-Synuclein at Serine-129 and we found that PLK2 levels were significantly increased (54%, N = 4, P < 0.05) in α-Synuclein injected brains compared to LacZ or parkin levels (Figure 5B &5E). Co-expression of parkin and α-Synuclein resulted in reduction of PLK2 levels.

Detection of phosphorylated α-Synuclein and decreased phosphorylation in the presence of parkin led us to examine protein phosphatases that may be involved in protein de-phosphorylation. No changes were observed with protein phosphatase-1 (data not shown) or the levels of the scaffolding subunit A (Figure 5F &5G) of protein phosphatase-2A (PP2A), in the presence or absence of either α-Synuclein or parkin or LacZ. Further examination of other sub-units of PP2A showed that parkin caused a significant (P < 0.05, N = 4) increase (~60%) in the protein levels of both the regulatory subunit B (PP2A-B) and the catalytic subunit C (PP2A-C) compared to α-Synuclein expression and LacZ (Figure 5F &5G). To verify that parkin mediates the decrease in α-Synuclein levels via PP2A, we inhibited PP2A with the classical inhibitor okadaic acid (OA) in the presence of parkin (Figure 5H). PP2A inhibition with OA prevented parkin from decreasing α-synuclein levels (Figure 1H, N = 6). These data indicate that parkin expression may specifically lead to changes in α-Synuclein de-phosphorylation via its effects on PP2A levels in MN9 D cells. To further test whether these effects are mediated by parkin expression, we expressed T240R mutant parkin (Figure 1I) and observed no difference in the level of α-Synuclein or PP2A levels, suggesting that parkin may mediate the phosphorylation of α-Synuclein via its effects on PP2A.

The increase in PP2A-B and C protein levels led to the examination of phosphatase activity in these animal models. Lentiviral parkin expression significantly increased (~60%, N = 4) phosphatase activity in parkin or parkin+α-Synuclein (Figure 5J) injected animals, compared to LacZ or α-Synuclein injected brains (Figure 5J). These data indicate that phosphatases, including PP2A and kinases, such as PLK2, may be involved in the modulation of α-Synuclein phosphorylation and parkin can activate PP2A, which may affect protein de-phosphorylation.

## Discussion

We generated gene transfer animal models and studied the effects of parkin on post-translational modifications of wild type α-Synuclein and examined the interaction between these two proteins. α-Synuclein expression caused cell death and increased PLK2 levels leading to α-Synuclein phosphorylation [18,35]. α-Synuclein expression increased GSK-3β activity and Tau phosphorylation. Parkin expression was associated with an increase in phosphatase activity. Parkin expression decreased the levels of PLK2 and phosphorylated α-Synuclein; and decreased GSK-3β activity and the phosphorylation of Tau. These data suggest that α-Synuclein over-expression increases kinase activity and leads to Tau and α-Synuclein phosphorylation, while parkin increases phosphatase activity to de-phosphorylate proteins and counteract the effects of α-Synuclein over-expression. While the effects of parkin on α-Synuclein are consistent with previous studies in mutant A30P α-Synuclein injected rats, which show that parkin reverses the effects of α-Synuclein on cell death [25], we further tested the role of parkin in post-translational modification of α-Synuclein, Tau phosphorylation and inflammation in the present models. The gene transfer animal model we developed demonstrates the pathological effects of wild type α-Synuclein. Lentiviral infection resulted in an increase in α-Synuclein levels in the striatum, within the pathological range of increase in PD, in which over-expression of α-Synuclein is associated with disease pathogenesis [36-38]. Expression of α-Synuclein resulted in cell death [25,39,40]. Inflammatory signs were observed in this α-Synuclein model and parkin protected against changes in astroglial morphology and inflammatory molecules, providing evidence of α-Synuclein-induced pathology and parkin suppression of α-Synuclein effects on inflammation. However, microglial activation may depend on a specific time course and localization of protein expression. Intracellular accumulation of α-Synuclein within the striatum might induce a different immune response compared to other brain regions [i.e. SN]. These results are consistent with previous data showing that parkin deficiency in mice increases the risk of inflammation and neuronal death [41].

Parkin expression results in α-Synuclein de-phosphorylation at Ser 87 and Ser 129 and attenuates the phosphorylation of Tau at several Ser and Thr residues. These findings are intriguing and suggest new mechanisms of α-Synuclein effects and parkin role in modulating phosphatase activity and its ability to recruit cellular substrates. The PP2A core enzyme comprises a scaffolding subunit A, a catalytic subunit B and a regulatory subunit C [42,43]. To gain activity toward specific substrates, PP2A-A interacts with variable regulatory B subunits to form a heteromeric holoenzyme [42,43]. Changes in phosphatase activity led to the examination of the relationship between α-Synuclein expression and endogenous Tau phosphorylation. Activation of the regulatory subunit PP2A-B mediates Tau dephosphorylation [44]. PP2A specifically targets Ser and Thr residues, resulting in protein de-phosphorylation [42,44]. The effects of parkin on PP2A and the decrease in phosphorylated α-Synuclein levels may explain the protective effects of parkin. However, it is not understood how parkin might affect PP2A activity or levels, we did not observe any direct interaction between the two proteins with co-immunoprecipitation (data not shown), and there are no previous reports to discuss any relationship between parkin and phosphatases. The association between parkin expression and decreased Tau and α-Synuclein phosphorylation is intriguing, and suggests that parkin may protect against α-Synuclein-induced cell death by maintaining a stoichiometric balance between phosphorylated and non-phosphorylated pools of proteins.

Modification of α-Synuclein at Ser 129 may trigger α-Synuclein pathology in dopaminergic cells, but only a fraction of α-Synuclein is phosphorylated [15]. p-Ser 129 appears to be the major phosphorylated form of α-Synuclein in LB inclusions [31,32]. Ubiquitinated p-Ser 129 increases in parkin-deficient mice [45] and increased levels of p-Ser 129 leads to protection of dopaminergic neurons in the presence of parkin [25]. However, we found that parkin decreased the levels of p-Ser 129 *in vivo*, and decreased the levels of higher molecular weight species (with no evidence of either ubiquitination or LB formation), suggesting that parkin expression may lead to a decrease in α-Synuclein level, primarily via reduction of its phosphorylated fraction. The data suggest that neither endogenous nor over-expressed parkin affects the native and p-Ser 129 levels of rat α-Synuclein [45,46], and the decrease in p-Ser 129 is only observed when human α-Synuclein is expressed. In cellular models of Synucleinopathies, ubiquitinated inclusions increase when human α-Synuclein is co-expressed with parkin and synphilin-1, but decrease when phosphorylation of α-Synuclein is blocked by expression of the S129A α-Synuclein mutant [33]. Taken together, the data suggest that phosphorylation may be an early cellular coping mechanism in Synucleinopathies, whereas ubiquitination of phosphorylated α-Synuclein is a later process involving possible sequestration and protein clearance.

p-Ser 87 is found in cell lines [11] and recent findings by Lashuel and coworkers demonstrated that p-Ser 87 co-localizes with α-Synuclein in LBs and is elevated in diseased brains [47]. The absence of p-Ser 87 from the rat brain suggests that this phosphorylated form is derived from expression of human α-Synuclein. Ser 87 lies within the hydrophobic non-amyloid component [48] region of α-Synuclein, and is one of the residues that distinguish the human α-Synuclein sequence from its mouse and rat homologs, suggesting that this residue may contribute to the differential modification of human and rat α-Synuclein. Moreover, α-Synuclein phosphorylation, particularly within the NAC domain (71-82) that promotes aggregation [49,50] and the C-terminus (120-140) that decreases aggregation [49], may alter the properties of α-Synuclein depending on the phosphorylation state of either Ser 87 or Ser 129. Further studies are required to determine if there is a crosstalk between the two phosphorylation sites of α-Synuclein.

## Conclusions

Taken together, these studies provide evidence that wild type human α-Synuclein is pathological and can be phosphorylated *in vivo *to result in protein aggregation. Parkin decreases GSK-3β activity and reduces Tau phosphorylation, inflammation and cell death. Phosphorylation of α-Synuclein in PD and other Synucleinopathies may be mitigated by the effects of PP2A. In this animal model, parkin protects against α-Synuclein-induced pathology by increasing phosphatase activity and decreasing PLK2 levels to attenuate the levels of aggregation-prone p-Ser-129 and p-Ser 87. Parkin expression may be used as a potential therapeutic strategy to counteract the effects of α-Synuclein toxicity in the Synucleinopathies.

## Abbreviations

M.O.I: multiplicity of infection; PD: Parkinson's disease; PP2A: phosphatase 2A; PLK2: Polo-Like Kinase-2; P-SER 129: α-Synuclein phosphorylated at serine 129; P-SER 87: α-Synuclein phosphorylated at serine 87; SN: substantia nigra; LBS: Lewy Bodies; LV-PAR: lentiviral parkin; LV-SYN: Lentiviral α-Synuclein; LV-LACZ: lentiviral LacZ.

## Competing interests

The authors declare that they have no competing interests.

## Authors' contributions

PJK performed experiments and prepared the manuscript. SBD collected some confocal images. LRF grew the MN9D_Syn _cells. KM-Z provided and plated the MN9D_Syn _cells. HL provided the Serine-87 antibody of phosphorylated α-Synuclein. GWR helped with critical evaluation of the data. CE-HM injected the animals, performed the experiments, analyzed the data and prepared the manuscript. All authors contributed to the reading and editing of the manuscript. All authors read and approved the manuscript.
